# The Role of Identification in Consumers' Evaluations of Brand Extensions

**DOI:** 10.3389/fpsyg.2018.02582

**Published:** 2018-12-17

**Authors:** Longinos Marin, Salvador Ruiz De Maya, Alicia Rubio

**Affiliations:** ^1^Marketing Department, University of Murcia, Murcia, Spain; ^2^Management Department, University of Murcia, Murcia, Spain

**Keywords:** consumer identification, brand extension, consumer behavior, brand alliance, brand fit

## Abstract

Brand extension is a widely adopted strategy for firms to take advantage of an existing brand's equity in a new product category. The main goal of this paper is to test the moderating role consumer-company identification plays in the effect of product fit and information on consumers' evaluations of brand extensions. Study 1 demonstrates the moderator effect of identification on the effect of category fit on consumers' purchase intentions for brand extensions and brand alliances. In Study 2, we proposed that identified consumers are not affected by information about the product, while low identified consumers rely more on that information. However, results show that the presence of information about the brand extension is only significant for identified consumers. For marketing managers, our results will help in decisions regarding extension category selection, segmentation strategy, and identification cuing.

## Introduction

Some firms launch new products under the format of brand extensions to take advantage of their brand's equity in a new category (Grime et al., 2002). Brand extension strategy implies the use of established and successful brand names to enter new product categories (Keller and Aaker, 1992). Firms widely employ this strategy because of beliefs that it builds and communicates strong brand positioning, enhances awareness and quality associations, and increases the probability of trials by reducing new product risk for consumers (Taylor and Bearden, 2002). Alternatively, firms use brand alliances to link with other firms or brands through their products or other aspects of their marketing programs. For example, Adidas has found success in using adhesive sports shoes with special rubber soles developed together with Goodyear (Adidas ranks 55th in the 2017 Interbrand ranking of most valued brands).

While the question of how companies benefits of brand extensions has been widely discussed and proved in the literature (Hayran and Gürhan-Canli, 2016), marketing managers still face the highly frequent problem of brand extension failures, which can reach rates between 80 and 90% in western countries, such as the United States (Batra et al., 2010). An especially relevant problem as brand extensions focus on categories away from that of the parent brand (Alexander et al., 2008). As such, if managers are able to identify a market segment strongly loyal or identified with the company, they can market the brand extensions to that segment and reduce the probability of failure.

Literature has already considered the determinants of consumers' brand extension evaluations, showing that category fit is crucial for success in brand extensions and alliances (Simonin and Ruth, 1998). New product evaluations are lower when its fit with the firm's skills is low, i.e., when the firm enter perceptually distant markets (Smith and Andrews, 1995). However, some companies are successful in launching new products with low fit with the parent brand. For example, the leisure company Virgin has successfully developed Virgin Health Bank to offer families the possibility of banking cord blood stem cells of their babies, apart from companies related to wine, pure water or megastore (www.virgin.com/company). In this sense, literature emphasizes the importance of identity similarity and attractiveness in shaping consumers' attitudes, preferences, and choices (Tajfel and Turner, 1985; Bhattacharya and Sen, 2003). However, extant literature has not provided a thorough understanding of how and when identification affects brand extension (Gammoh et al., 2006).

The main goal of this paper is to test the moderating role that CCI plays in the consumers' evaluations of brand extensions. We also extend this reasoning to brand alliances. In addition, we also test that moderating effect when consumers are exposed to the brand extension. Expanding the results of Rubio and Marin's (2015) research, three arguments justify the influence of consumer identification. First, a more positive brand attitude is positively related to consumer's intention to purchase the brand's extension (Aaker and Keller, 1990). In addition, consumer's associations related to the benefits of the brand in terms of self-expression and value-expression play a significant role in brand extension and brand alliance evaluations (Köstring and Blümelhuber, 2007; Rubio and Marin, 2015). Third, CCI leads to consumers' extra and positive behaviors (e.g., promotion, participation, and recruitment) that support companies (Bhattacharya and Sen, 2003).

In the remaining sections, we review the literature and propose hypotheses regarding the effects of identification on the purchase intention of brand extension. Study 1 is based on a field experiment to test the effects category fit and brand strategy on identification. In study 2, we use an experiment to test how identification moderates the effect of information about the new product on brand extension success. Finally, we discuss the theoretical findings and managerial implications, including an outline for a future research agenda in the area of consumer identification with companies.

## Literature Review and Hypotheses

### The Company-Consumer Identification

Bhattacharya and Sen (2003, p. 76) define company-consumer identification (CCI) as “the primary psychological substrate for the kind of deep, committed, and meaningful relationships that marketers are increasingly seeking to build with their customers.” A self-definition stable and secure constitutes a basic need, which can explain the consumer's interest in identifying themselves with groups and organizations (Erez and Earley, 1993). But more specifically, and following the Social Identity Theory, self-definitions are combinations of relevant social identities (e.g., education, job, region, or county of origin, etc.) adn idiosyncratic attributes (Tajfel and Turner, 1985).

In addition to Social Identity Theory, organizational identity can also contribute to explain the process involved in consumers' identification with organizations (Pratt, 1998). Organizational identification refers to the “degree to which individuals feel a sense of connectedness with an organization” (Mael and Ashforth, 1992) in such a way that the attributes they perceive define the organization are similar to those that define themselves (Dutton et al., 1994).

Bhattacharya and Sen (2003) specifically referred to consumers when analyzing how people identify with organizations. They proposed the concept of consumer-company identification based on, what, they suggest, are the five consequences of identification: loyalty, promotion of the company, recruitment of new customers, resilience to information that may negatively influence the company, and strong claim on company. Brashear-Alejandro et al. (2016) corroborated this behavior demonstrating that feeling of status and belongings are benefits associated with loyalty programs that contribute to consumers' identification with the organization. Empirical research has also confirmed some of the consequences of identification, such as loyalty, product utilization and extra role behaviors as providing helpful information to other customers (Marín et al., 2009). As Social Identity Theory (Tajfel and Turner, 1985) posits, identification causes people to become psychologically attached to the company and expend voluntary effort on its behalf. But identified consumers' support of the company is not likely to be restricted to consumption (Ahearne et al., 2005), on the contrary, identification will favor the development of extra-role behaviors.

Some studies indicate that brand identification may better reflect consumers' connection with companies(Lam et al., 2013; Tuskej et al., 2013), which make sense since the best examples of identification are associated to companies whose name matches the brand name (e.g., Apple). This perspective of brand identification is related to literature that focuses on consumer commitment (Choi and Ahluwalia, 2013), as it expressed a desire to keep a long term relationship with the brand (Brown et al., 2005) that will be mutually beneficial. Recently, Tuskej et al. (2013) demonstrate that identification with the brand exerts a positive influence on consumer's commitment to maintain meaningful relationship with the brand.

It is also of interest to point out that while consumer-company identification and ownership may influence similar variables such as evaluations or purchase intentions (see Kirmani et al., 1999; Bhattacharya and Sen, 2003), the two concepts are clearly different. Ownership is the result of acquiring the brand voluntarily acquisition, having a direct experience, or having the physical possession (Kirmani et al., 1999) and, therefore, it refers to the possession or the right to use it. Identification, in contrast, is defined as the individual's perception of the degree he/she shares with the company the same defining attributes, which are different from those of individuals who do not belong to this group formed around the company (Pratt, 1998). Ownership and identification are different (a consumer may be identified with a company without owning a product of that company and vice versa), but there is a correlation between the two concepts (Bartsch et al., 2016).

### Brand Extensions

A brand represents a category in consumers' mind that has a dominant attribute, associations that contribute to its image, and a related attitude (Boush and Loken, 1991). It captures images that consumers have formed through the acquisition of information and experiencial interactions with the brand (Swait et al., 1993). Through brand extensions, the company takes advantage of marketplace growth opportunities and exploits positive brand equity (Martinez and Pina, 2010).

Corporate branding strategies are part of the firm's product decision (Gürhan-Canli and Batra, 2004) whose synergies with the other marketing decisions contribute to the firm assets (e.g., Aaker and Keller, 1990). Companies communicate and launch new products through one of the following three brand actions: using the parent brand to make use of that brand value, using a new brand name different and separated to the parent brand name, and using both the new and the parent brand names (Berens et al., 2005).

Individual differences between consumers, such as chronic or situational motivation, ability and opportunity to process extension information significantly influence how consumers perceive and evaluate brand extensions (Keller, 2016). Among these individual difference factors, consumers' identification with the company is of high interest because it does not only refer to motivation related to product acquisition, but also to motivation to keep themselves linked to the company.

Attitudinal and behavioral commitments are two forms of maintaining the link with the company and, as such, they are likely outcomes of identification and help reinforce the strength of identification (Einwiller et al., 2006). Identification with a company also results in a commitment to the company (Bergami and Bagozzi, 2000), implying attitude strength, repeat buying, and loyalty. The effects of CCI are persistent and very effective at immunizing customers against competitive actions and keeping them linked to the company (Haumann et al., 2014). Therefore, following Rubio and Marin (2015), highly identified consumers will more likely buy a new product launched by the company under a brand extension than those consumers who identify less with the company.

*Hypothesis 1: When exposed to a brand extension, consumers who strongly identify with the company will show higher purchase intention than consumers who weakly identify with the company*.

### The Moderating Effect of CCI

Categorization theory embraces that when subjects use categories to arrange information and objects are more efficient in processing and understanding their own environment (Rosch and Mervis, 1975). The association of a product or object to a category implies that the subject transfer to the product or object the attitude toward the category and its components (affect and cognitions). A brand extension involves the introduction of a new object (the extension) into the category defined by the brand (products marketed under that brand). In these brand extension decisions, the ability of a well-known brand to reduce the uncertainty about a particular extension category lies primarily in the fit between the brand and the category (Smith and Andrews, 1995; Laforet, 2008). By *fit*, we mean the degree of similarity between a product extension and existing products affiliated with the brand (DelVecchio and Smith, 2005), that is, we refer to category fit.

Regardless of how fit is conceptualized, as it increases consumers transfer their favorable associations with an established brand to the brand's extension more confidently. In that context, fit contributes to more positive evaluations of the brand extension (Aaker and Keller, 1990), a lower probability of negative evaluations which will be also less severe (DelVecchio, 2000).

However, additional insights into how category fit and purchase intention relates can be gleaned when considering consumer identification. But behind the positive outcomes of identification, such as loyalty and resilience to negative information about the company (Einwiller et al., 2006), lies identified consumers' motivation to look for a positive identity and self-esteem (Bhattacharya and Sen, 2003). The purchase of a brand extension provides consumers with ways to interact with the company, reinforcing positive identity and self-esteem. Therefore, identified consumers will buy new products the company launches under the form of a brand extension, whatever is the fit these products.

On the contrary, non-identified consumers will show positive brand associations because of their previous exposure to the brand and the fact that has been chosen in previous decisions over other brands. However, because these consumers are not as strongly linked to the company as identified consumers are, their reactions to brand extensions will be affected by other variables such as fit (Aaker and Keller, 1990; Rubio and Marin, 2015). Thus, for high category fit brand extensions, consumers will transfer their positive associations with the brand to form positive evaluations of the new extension, while this transference will not occur for low category fit brand extensions. Therefore, we propose the following:

*Hypothesis 2: Consumer-company identification moderates the effect of* category *fit on purchase intention of brand extensions*.

*H2a: When exposed to a brand extension, consumers who strongly identify with the company will show similar purchase intention for high and low levels of* category *fit*.

*H2b: When exposed to a brand extension, consumers who weakly identify with the company will show higher purchase intention for the brand extension with high* category *fit than for the brand extension with low* category *fit*.

### Brand Alliance, as an Alternative to Brand Extension, and the Moderating Effect of CCI

When companies label new products with brand alliances, the resulting joint brand combines proprietary assets from the two brands (Simonin and Ruth, 1998), in an effort to obtain synergies from their marketing cooperation. The main goal of this collaboration is the As such, brand alliances are a useful extension strategy because they strengthen the attribute profile of the extension (Park et al., 1996), help the partner brands gain advertising synergies, and improve customers' attitudes toward the parent brands (Simonin and Ruth, 1998). For example, the alliance involved in the jointly branded credit card American Airlines-Visa allows the accumulation of frequent flier miles to be used on American Airlines flights with all purchases made on the Visa card.

Although by definition a brand alliance consists of two partner brands, in general, both brands do not contribute equally to the co-branded concept (Kumar, 2005). Typically, one of the two brands serves as a dominant or head brand, while the other serves as a dominated brand (Murphy, 1988). For identified consumers, the brand alliance will be considered a composite concept dominated by the brand they identify with, as they will have more interactions and, therefore, will show more attraction for that brand (Marín et al., 2009).

The organization's goals are a significant commitment for identified consumers, motivated to voluntarily dedicate efforts to support it. As such, highly identified consumers will be committed in a brand alliance to continue buying the company's products to maintain their links with the organization (Ahearne et al., 2005). However, consumers with a low identification do not feel the need to keep their contact with the company, and other variables will drive their purchase intention. For that reason, it is important the brand alliance product show a high category fit with part or all of the established company's product portfolio, if the company wants to transfer relevant associations from the established constituent brand to the brand alliance product (Park et al., 1996). Therefore, consumers with a low identification will be affected by category fit. Thus:

*Hypothesis 3: Consumer-company identification moderates the effect of category fit on purchase intention of brand alliances*.

*H3a: When exposed to a brand alliance, consumers who strongly identify with the company will show similar purchase intention for brand alliances with high and low category fit*.

*H3b: When exposed to a brand alliance, consumers who weakly identify with a company will show higher purchase intention for brand alliance with high category fit than for brand alliance with low category fit*.

**Figure 1:**
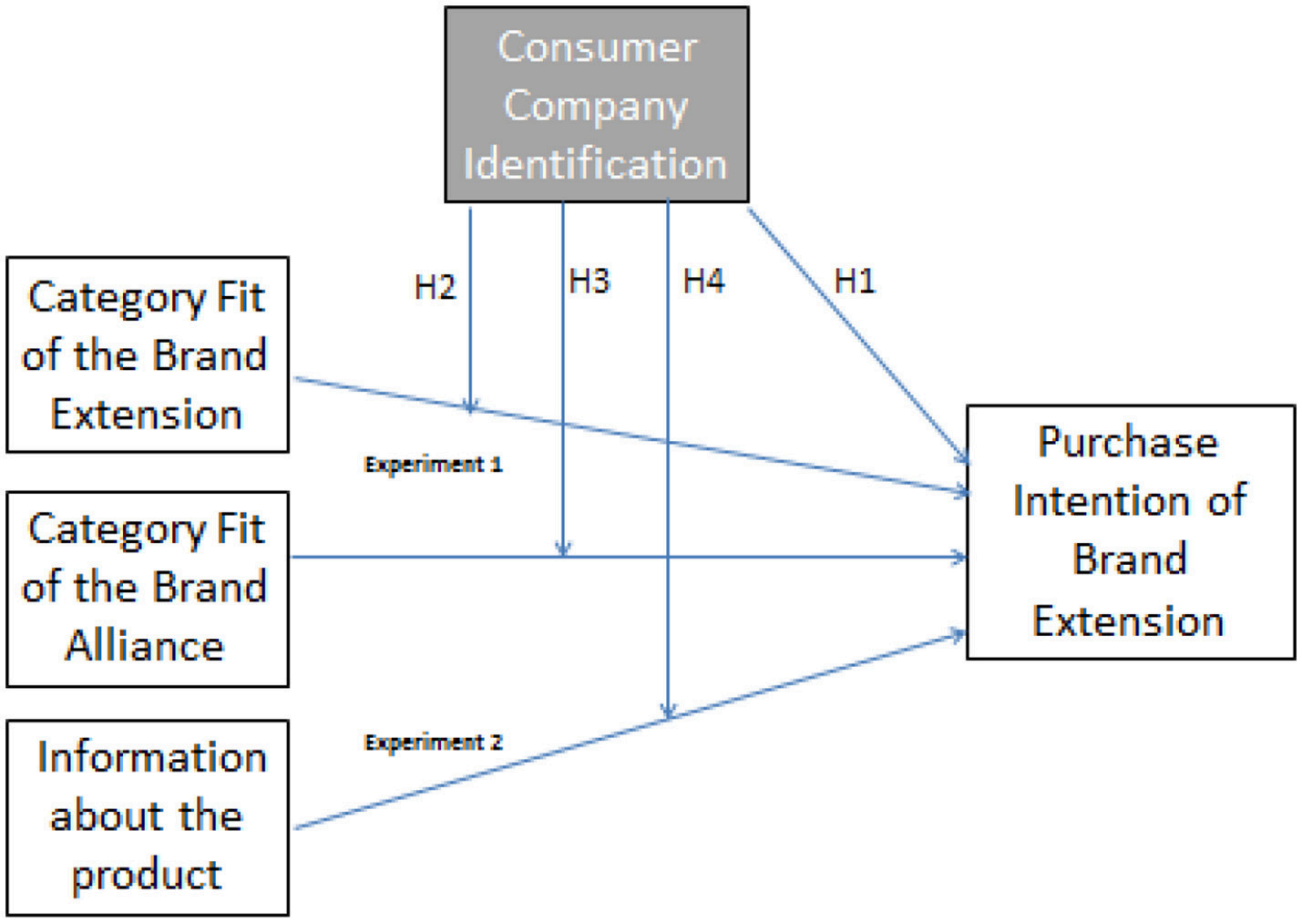
Framework proposed.

Hypotheses 1 to 3 are part of the proposed final model displayed in Figure 1.

## Study 1

We collected data through a field experiment. The respondents were consumers of a large bank and we asked them to collaborate in the evaluation of new products the company was planning to launch.

### Procedure and Stimuli

A 2 × 2 between-subjects experimental design allowed us to manipulate (1) brand decision (extension vs. alliance) and (2) category fit (insurance as a high fit product vs. travel, the low fit product). Data were collected through personal interviews carried out by a professional interviewer. Subjects were customers of a bank responsible for their own and/or their family financial matters, Interviewers contacted them just before entering the bank's branch and assigned them at random to one of the four experimental conditions. Prior to participation in the study, we informed subjects about the academic purpose, the general goal, the guarantee of their anonymity, and that they could withdraw at any time. We carried out the study in accordance with the recommendations of institutional and national guidelines and regulations (at the time of the data collection, ethics approval was not required). All subjects gave written informed consent.

Each respondent evaluated one of the four products after being exposed to the corresponding leaflet. To ensure sufficient realism of the materials, we based the contents of the leaflets, printed by a company dedicated to designing advertising material, on existing print advertisements (images were provided by the company) and brands. We conducted open interviews with professionals and used a pre-test to choose the products that best allowed us to manipulate *category* fit in the context of a financial services company: insurance (high fit) and travel (low fit).

Whether the product was a brand alliance or a brand extension was clearly presented to respondents in the leaflet. For the brand alliance, the two versions of the leaflet described the product showing the two logos of the financial company and either the insurance company or the travel agency. The two leaflets for the brand extension alternatives (insurance and travel) only included the brand of the financial services company.

After an introduction and collecting demographics, we exposed the respondents to the brochure. We then asked them about their purchase intention, identification with the company, how they perceived the new product fitted the financial service category (the parent product), attitude toward the financial sector, and attitude toward the allied brand (only for the two brand alliance conditions). In total, 380 clients of the bank participated in the study (ninety five per condition). The average age of the sample was 38 years, and men accounted for 51.3%. Most subjects had a university degree (52%) and, on average, they had been customers of the financial company for 12.3 years.

### Measures

We assessed measures of attitudes toward each partner brand and toward the financial sector through seven-point bipolar semantic differential scales taken from Simonin and Ruth (1998) (negative/positive, unfavorable/unfavorable, bad/good) The measurement of *category* fit (complementary, substitutability) was adapted from Aaker and Keller (1990). We measured purchase intention with the three-item scale from Grewal et al. (1998), used by Taylor and Bearden (2002) in a brand extension context. Finally, we combined Bergami and Bagozzi's (2000) item to measure the consumer's identification with a company with Ahearne et al.'s (2005) visual and verbal identification scale.

We evaluated constructs reliability and validity through confirmatory factor analysis (CFA). Overall, the measurement model's fit statistics showed reasonable values. In the completely standardized solution, indicators clearly loaded on their corresponding factors (Table 1), providing evidence of the indicators' validity. Moreover, composite reliability indexes showed higher values than the recommended level of 0.6 (Bagozzi and Yi, 1988).

**Table 1:** Study 1. Results of the CFA.

**Items**	**Mean (S.D.)**	**λ_**c.e**_ (t)**	**Reliability**
**PURCHASE INTENTION (PI)**			
If I were going to buy car insurance (cruise), the probability of buying this one is very high	3.83 (1.74)	0.94 (24.54)	CR = 0.94 AVE = 0.85 Alpha = 0.94
The probability that I would consider buying this car insurance (cruise) is very high	3.70 (1.75)	0.93 (24.09)	
The likelihood that I would purchase this car insurance (cruise) is very high	3.97 (1.86)	0.88 (21.67)	
**CONSUMER COMPANY IDENTIFICATION (CCI)**			
Visual scale (Bergami and Bagozzi, 2000)	3.95 (1.90)	0.91 (16.29)	CR = 0.70 AVE = 0.55 Alpha = 0.73
Indicate the degree to which your self-image overlaps with X‘s image	3.29 (1.74)	0.54 (10.12)	
**CATEGORY FIT (FIT)**			
The “complementarity” of the financial services and insurance services (cruises) is very high	3.55 (1.89)	0.60 (8.24)	CR = 0.77 AVE = 0.64 Alpha = 0.76
The substitutability of the financial services and insurance services (cruises)is very high	4.46 (1.68)	0.92 (9.24)	
**ATTITUDE TOWARD THE FINANCIAL SECTOR (ATF)**			
My attitude toward financial sector is positive (negative)	4.75 (1.65)	0.98 (26.24)	CR = 0.98 AVE = 0.97 Alpha = 0.99
My attitude toward financial sector is favorable (unfavorable)	4.77 (1.64)	0.99 (26.46)	
My attitude toward financial sector is good (bad)	4.78 (1.65)	0.98 (26.27)	
**ATTITUDE TOWARD THE ALLIED BRAND**^**a**^			
My attitude toward this brand is positive (negative)	4.25 (1.42)	0.91 (22.24)	CR = 0.93 AVE = 0.91 Alpha = 0.98
My attitude toward this brand is favorable (unfavorable)	4.17 (1.39)	0.93 (26.05)	
My attitude toward this brand is good (bad)	4.48 (1.55)	0.89 (19.22)	
Goodness-of-fit statistics^b^			
χ^2^(29) = 68.42, *p* < 0.00; AGFI = 0.93 GFI = 0.97 CFI = 0.99; RMSEA = 0.06, NNFI = 0.98			

a *Results for the 190 observations of the two brand alliance conditions*.

b *Matrix results with the 380 observations, excluding the variable Attitude toward the allied brand*.

*CR, composite reliability; AVE, average variance extracted; Alpha, Cronbach's alpha*.

For scale validity, we checked convergent and discriminant validity. First, all the parameters of the indicators were statistically significant (t > 1.96) and > 0.70 (except for the second item of identification which at least is > 0.50), which shows convergent validity (Anderson and Gerbing, 1988). In addition, we checked that for each latent variable in the phi matrix, the average variance extracted exceeded the square of its correlation with the rest of the latent variables (Table 2), which according to Fornell and Larcker (1981) indicates discriminant validity.

**Table 2:** Discriminant validity.

	**PI**	**CCI**	**ACTEFIN**	**AVE**
PI	1.00			0.85
CCI	0.37	1.00		0.55
ATF	0.50	0.33	1.00	0.97

*Square of the correlation of the variables and AVE*.

### Results

We averaged the two items of the category fit scale; the means of this variable show that the category fit manipulation worked as expected. Respondents in the travel condition perceived less category fit (M_LOWFIT_ = 3.15) than those in the insurance condition (M_HIGH FIT_ = 5.41; [*F*_(1, 378)_ = 523.1, *p* < 0.01]. In addition, we conducted a median split to separate high and low identified respondents, based on an averaged measure of the two items. The mean score of identification was significantly different between the two groups [M_LOW IDE_ = 2.31, M_HIGH IDE_ = 5.02; *F*_(1, 378)_ = 858.58, *p* < 0.01].

Table 3 reports the mean scores for purchase intention. In addition to the three independent variables (brand strategy, category fit, and CCI), we added attitude toward the financial sector as a covariate, to account for its effects through ANCOVA (Table 4). The effect of the covariate was significant, which justified including it in the analysis, as was the effect of CCI [*F*_(1, 371)_ = 167.59, *p* < 0.01], which confirmed H1. Highly identify subjects' purchase intention (M_HIGH IDE_ = 4.91) was higher than that of low identified consumers (M_LOW IDE_ = 2.84) for both brand strategies (extension and alliance). A significant main effect of category fit [*F*_(1, 371)_ = 16.46, *p* < 0.01] also indicated higher purchase intention for the high fit product (M_HIGH FIT_ = 4.23) than for the low fit one (M_LOWFIT_ = 3.44). Neither the main effect of brand strategy [*F*_(1, 371)_ = 0.24, *p* < 0.61] nor any of its interactions were significant. However, there was a significant interaction effect of CCI and category fit [*F*_(1, 371)_ = 14.83*, p* < 0.01]. As Table 3 shows, for highly identified consumers, purchase intention was always high, for products with both high (M_EXT_ = 4.84, M_ALLI_ = 5.01) and low (M_EXT_ = 5.02, M_ALLI_ = 4.84) category fit. In contrast, for weakly identified consumers, purchase intention was higher for products with high (M_EXT_ = 3.53, M_ALLI_ = 3.41) than for products with low (M_EXT_ = 2.32, M_ALLI_ = 2.29) category fit. The three-way interaction was not significant; thus, the results for the identification–fit interaction held for the brand extension and the brand alliance, in support of H2 and H3.

**Table 3:** Study 1. Mean scores for purchase intention.

**Brand strategy**	**Low category fit**		**High category fit**	
	**Low identification**	**High identification**	**Low identification**	**High identification**
Brand extension	2.32	5.02	3.53	4.84
	(1.14)	(1.62)	(1.53)	(1.21)
Brand alliance	2.29	4.84	3.41	5.01
	(1.20)	(1.49)	(1.66)	(1.47)

**Table 4:** Study 1. ANCOVA results for purchase intention.

**Effect**	***F***	***P***
Attitude toward the financial sector	5.81	0.01
Category fit (Fit)	16.46	0.00
Consumer company identification (CCI)	167.59	0.00
Brand extension vs. brand alliance (BEvBA)	0.24	0.61
Fit × CCI	14.83	0.00
Fit × BEvBA	0.08	0.93
CCI × BEvBA	0.01	0.93
BEvBA × Fit × CCI	0.78	0.38

The availability of attitudes toward the allied brand for half the sample that was exposed to the two brand alliance conditions allowed including this variable in the analysis for these 190 respondents. The results (Table 5) showed parallel results to the previous analysis: main effects of CCI [F_(1, 189)_ = 58.45, *p* < 0.01] and category fit [*F*_(1, 189)_ = 9.72, *p* < 0.01], and a significant interaction of the two variables [*F*_(1, 189)_ = 6.01, *p* < 0.01]. First, a significant effect of the covariate attitude toward the allied brand [*F*_(1, 189)_ = 17.62, *p* < 0.01], which shows that the higher the attitude toward the allied brand, the higher the purchase intention (correlation = 0.46, *p* < 0.01). These results confirm H3 even when we account for attitudes toward the allied brand.

**Table 5:** Study 1. ANCOVA results for purchase intention in the brand alliance conditions.

**Effect**	***F***	***p***
Attitude toward the financial sector	0.71	0.40
Attitude toward the allied brand	20.52	0.00
Category fit (Fit)	9.75	0.00
Consumer company identification (CCI)	82.19	0.00
Fit × CCI	6.20	0.01

## Study 2

Prior research has shown that the effect of category fit on consumers' acceptance of brand extension may be moderated by variables such as product ownership (Kirmani et al., 1999), consumer's mood (Barone et al., 2000) or the number of products associated with the brand (DelVecchio, 2000). In study 1 we contribute to that literature demonstrating that the relationship between the consumer and the company (identification) also shows a significant moderation effect. However, if we take into account that product category fit is a diagnostic cue used by consumers to make inferences when deciding about a new product introduced as a brand extension (Klink and Smith, 2001), information about the product will show similar effects, i.e., it will be used to make inferences when deciding about the purchase of brand extension. But what we do not know is whether the moderating effect of consumer company identification not only holds for product category fit, but more generally it moderates the effect of available information about the new product introduced as a brand extension on consumer purchase intention. The goal of study 2 is to fulfill that gap in the literature.

### The Effect of Information About the Product

The information that consumers access concerning an existing product serves as a main source to evaluate it, contributing to higher information processing and attitudes (Sicilia and Ruiz, 2010). However, when there is not enough information, consumers retrieve from memory accessible diagnostic cues such as category, price, brand name, or product warranty to make inferences that can fill in the gap (Simmons and Lynch, 1991).

Identification with the company is positively related to information accessibility because identified stakeholders maintain more interactions with the company (Bhattacharya and Sen, 2003) and, as such, they access and retrieve more favorable and relevant information (Scott and Lane, 2000). They also have more information about the company and its products, even about the new products, than non-identified consumers. In addition, identified consumers show in-role (loyalty and positive word of mouth) and extra-role behaviors (participation, defense of the company, etc.) based on their motivation to maintain their link with the company (Ahearne et al., 2005). This motivation will lead them to higher purchase intention of the brand extension whether it shows high or low category fit, as a new avenue to continue or increase their relationship with the company. Based on this reasoning, we propose:

*H4a: When exposed to a brand extension, consumers who strongly identify with the company will show similar purchase intention for the brand extension with information about the product and for the brand extension with no information about the product*.

*H4b: When exposed to a brand extension, consumers who weakly identify with the company will show higher purchase intention for the brand extension with information about the product than for the brand extension with no information about the product*.

### Procedure and Stimuli

Following Klink and Smith's (2001) procedure, we held a meeting with four brand experts to identify brands that were (1) reputable, (2) established, (3) working with different product categories (and, therefore, with different category fits), and (4) with identified and non-identified consumers. While they provided a list of 12 brands (both national and international), they also agree that Apple is a very well-known brand which also provides many possibilities to build the stimuli for the experiment. We also asked 10 subjects to mention products that may likely be new lines of product implemented by Apple. Through the evaluation of the realism and likelihood associated to the idea that Apple could launch those products, we selected a set of speakers. The stimuli were built using images from real products.

We used an experimental design with one variable manipulated between-subjects, information about the product (present vs. absent), while the second independent variable, identification, was measured. The stimulus (ad) with no information about the product did not include any description of the product, showing only the picture of the product, the Apple logo and the price. We used an *ad-hoc* composition to create a fictitious ad with the image of a real product downloaded from a website. In the other version of the ad, a paragraph with a product description like those used by companies in websites was included at the bottom (see Appendix). The price was the same across the two stimuli.

One hundred and fifty five observations were collected through Amazon MTurk in USA. Age range from 22 to 65 years old, with mean 38.8 and 40% women. Prior to accessing the questionnaire, we informed participants about the general goal, the academic purpose, the guarantee of their anonymity, and that they could withdraw if they do not accept the instructions provided to complete the questionnaire: answer all questions with honesty, not perform other activity, and not spend more than 15 min. Data collection was approved by the ethical committee of the University of Murcia. Consent to participate in the study was obtained through a click to access the questionnaire and instructions to abandon the study if participants were not comfortable with the content and other instructions. Participants received $1 for completing the study.

When entering the questionnaire, subjects were randomly assigned to one of the two experimental conditions. First, they were informed of the purpose of the study (evaluation of a new product) and, then, exposed to the ad. After, they answered questions about their purchase intention of the product, attitude toward technology, age, gender, their identification with Apple and one item to check the manipulation of additional information about the product (the ad included detailed information about the product). Purchase intention, attitude toward technology and identification were measured with the same scales used in Study 1, with seven point scales, also applied to the manipulation check item. Values of Cronbach's alpha were over.7 (Purchase intention = 0.90, Attitude toward the technology = 0.97, Identification = 0.85).

### Results

With the item introduced in the questionnaire to check the manipulation, we showed that it worked as expected. The agreement with the item was higher for subjects in the presence of information condition than for subjects in the no information condition [Mean for presence of information = 6, Mean for absence of information = 2.21; *F*_(1, 153)_ = 262.83, *p* < 0.01].

Then, we ran a regression to test the effect of additional information, identification, and their interaction on purchase intention. We also mean centered the two variables involved in the interaction to reduce multicollinearity. Attitude toward technology, age and gender were introduced as control variables. Results show that while none of the control variables exert a significant influence on purchase intention (b attitude toward technology = 0.11, *p* < 0.09; b age = 0.00, *p* < 0.76; b gender = 0.04, *p* < 0.88), the effect of the two main variables and their interaction were significant (b additional information = 0.20, *p* < 0.00; b identification = 0.47, p < 0.00; b interaction = 0.07, *p* < 0.02). The b of the intercept was 2.36 (*p* < 0.00).

**Figure 2:**
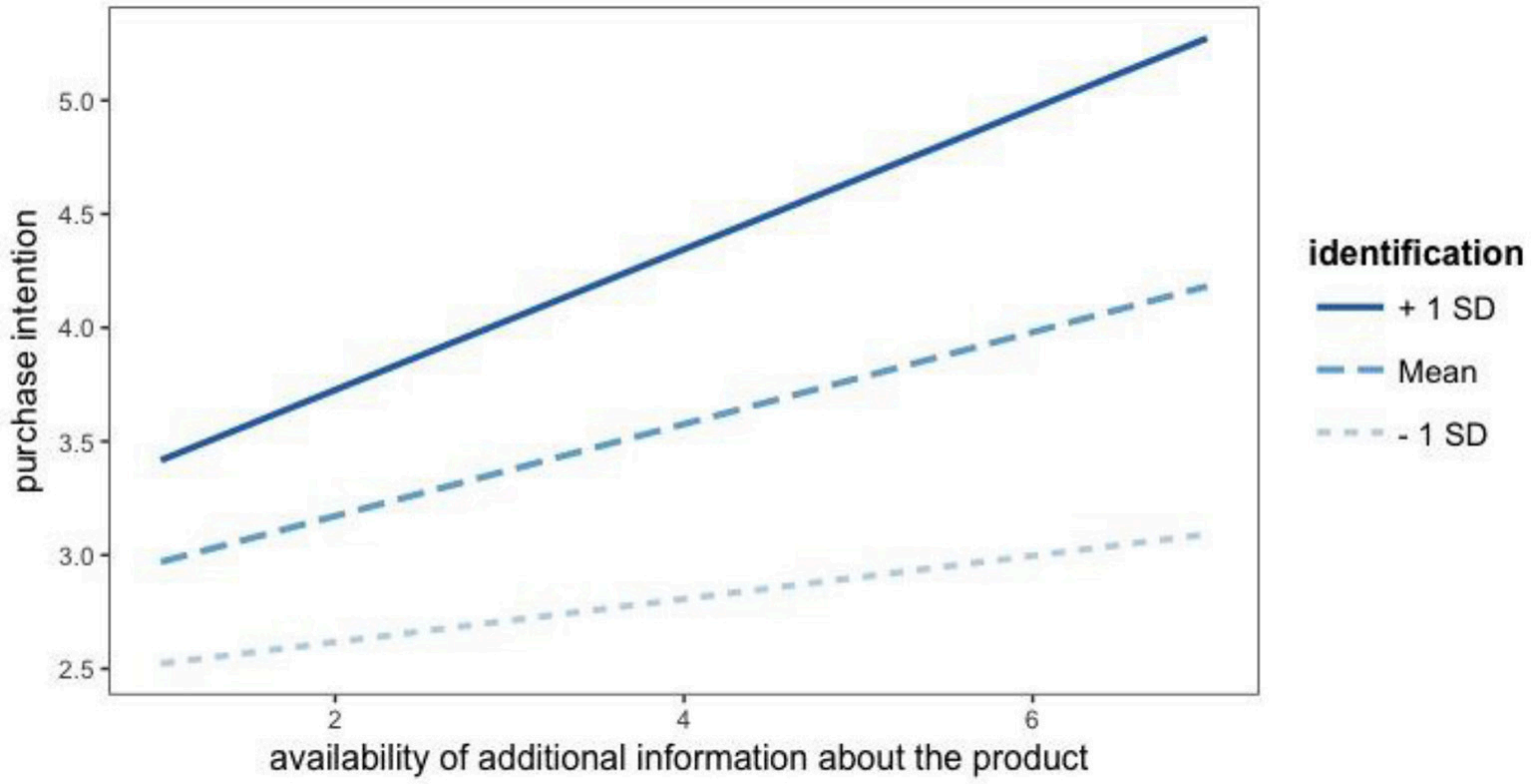
Effect of additional information about the product and identification on purchase intention.

We used floodlight analysis through the Johnson-Neyman's procedure (Spiller et al., 2013) to further analyze the interaction. This approach gave us the range of values of identification for which the perception of additional information influences purchase intention. Results, obtained with the probemod R package (Tan, 2015), show that the effect of perceived additional information is positive and significant (i.e., confidence interval does not contain zero at *p* = 0.05) for values of identification above 2.01. In other words, for subjects with a very low identification (32% or our sample) the availability of additional information about the product does not contribute to increase their purchase intention (b = 0.09, *p* < 0.16 when identification = 1.69, i.e., at the point of mean – 1 sd; Figure 2). Only for subjects with values of identification above 2.01 the availability of additional information shows greater levels of influence on purchase intention, as the identification increases (b = 0.31, p < 0.00 when identification = 4.87, i.e., at the point of mean + 1 sd; Figure 2).

## Discussion and Conclusions

This paper shows that the relationship consumers keep with the company can influence the success of brand extension activities. Whereas, previous research focuses on product features, such as category fit, attitudes and information (Simonin and Ruth, 1998) as critical drivers in explaining consumers' reactions to brand extensions, the focus here is on the significant role played by consumer-company identification.

While extra-rol behaviors (e.g., customer recruitment) and in-role behaviors (e.g., loyalty) have been traditionally associated with outcomes of consumer's identification (Ahearne et al., 2005; Einwiller et al., 2006), our research shows an additional consequence, that is, the patronizing of the brand extension activities of the company. This conclusion derived from study 1 is based on the idea that identified consumers are not affected by brand-extension fit, the key influential factor in assessing extensions (Hayran and Gürhan-Canli, 2016). Brand extension products with both low and high fit with the parent brand (low consistency or low overlapping of attributes) are highly prefer by identified consumers, as they offer new avenues to keep their relationship with the company. Our findings contribute to the brand strategy literature as they confirm a moderator effect of identification on the effect of category fit on consumers' purchase intentions. Thus, we offer an alternative explanation for the phenomenon of success of products with low fit with the parent brand launched as brand extensions.

Moreover, our research shows that this effect of identification is also valid for brand alliances. When a company launches a product under a brand alliance, identified consumers also show high purchase intention independently of the product category fit. These findings complement the argument of previous research about the advantages of selecting the allied brand taking into account its perceived quality and reputation (Rao and Ruekert, 1994), as a way to guarantee reciprocal positive effects for the partner brands (Simonin and Ruth, 1998). In fact, if we consider the segment of identified consumers, the positive effect of identification also holds for the brand alliance, that benefits from the unconditional loyalty of that segment. Definitely, nowadays consumers consider brands as products attributes to which they associate a certain capacity to generate functional and emotional benefits, instead of just identifiers of particular products (Gómez-Suárez et al., 2017).

Contrary to expected, highly identified consumers are more affected by information about the product than weakly identified consumers, as demonstrated with study 2. While it is clear that the former are highly interested in purchasing the products of the company, brand extensions too, our results have shown that they are also interested in processing the information about the new products the company provides. It seems they are highly motivated to read and correctly perceive the information about the product and the brand. According to the accessibility-diagnosticity model, accessible information is not used as an input for judgment and choice when more diagnostic or probative information is available (Feldman and Lynch, 1988). A piece of information is perceived as diagnostic if it helps the consumer assign a product to one (and only one) cognitive category. In contrast, information that is ambiguous or that implies multiple possible categorizations is non-diagnostic. For that reason, it is more likely that consumers use diagnostic inputs than non-diagnostic, as the latter imply ambiguous or multiple categorizations and, therefore, an increase in the complexity of the decision. Therefore, it is possible that identified consumers process the brand extension information as diagnostic and the purchase intention increases with the additional information provided.

In addition to the contributions to consumer behavior literature, our research holds managerial implications for decisions related to brand extensions, segmentation strategies and communication activities. While companies can launch new products through brand extensions or brand alliances, to benefit from the brand image, they should previously measure to what extent consumers are identified in the target segment. If the proportion of identified consumers is high, they do not need to pay much attention to category fit, a key determinant of brand extensions success. Managers can also target only identified consumers to minimize the risk of failure in the first stages of the new product launching.

However, when the identification of the target segment is low, managers should consider the category fit associated to the brand extension. High category fit will contribute to higher purchase intention. And if the category fit is low, they still can reduce the negative effect of this variable by communicating information about the new product. Consumers will process this information to build their purchase intentions while inferences based on the category fit will be less relevant.

Marketing managers should also take into account that while consumer-company identification plays an important role at the introduction stage of the new product launching, brand positioning significantly contributes to growing identification over time (Lam et al., 2013). Therefore, companies would also benefit of implementing communication activities that leverage the relevance of the brand as a social identity. Specially those that follow an “exploit brand equity” strategy, labeling their products with the corporate brand in every market they operate (Dawar and Anderson, 1994).

Our research is also affected by some limitations. First, our sample includes only customers from a single company, which limits the generalization of the results to customers who simultaneously operate with more than one financial services provider. Second, brand extensions and alliances in other industries may be affected by other organizational variables that given our single industry context we did not use. Third, a new brand (as a third alternative to brand extension and brand alliance) would have provided a better understanding of brand strategies in the context of identified consumers. This could be an interesting avenue for future research. Finally, other variables such as communication strategy and consumer-brand engagement may influence consumers' behavior and should be considered in future research too. Recent literature has shown how consumer engagement with the brand influence consumers in interactive virtual environments (e.g., Lafferty et al., 2016) where companies are nowadays promoting their brand extensions too. Given the profound changes taking place in markets, it is necessary to pay attention to how the consumer-brand relationships continue to evolve. Among future trends, there should be considered those that are likely to have a greater impact on these relationships, such as the opportunities offered by an efficient management of Big Data and the advent of Marketing 4.0 (Gómez-Suárez et al., 2017).

## Author Contributions

LM and SRDM conceived and drafted the paper and analyzed the data. AR reviewed related studies. All authors wrote, reviewed, and commented on the manuscript.

### Conflict of Interest Statement

The authors declare that the research was conducted in the absence of any commercial or financial relationships that could be construed as a potential conflict of interest.

## Appendix

Paragraph with the product description used in the “information about the product: present” condition of study 2.

*5.1-Channel System with 1000W Output* + *Built-in Wi-Fi. Experience the next generation of our groundbreaking Smart Home Theater with professional sound quality in a powerful and ultracompact device to enhance the Home Theater experience. Take your 3D viewing experience to a whole new level. 3D Sound Plus synchronizes the sound to match the motion and depth of the image, while giving you a fully immersive 3D experience. Hear your favorite music for the first time with the innovation of analog and digital sound combined. It creates a truer, more immersive sound that digital alone cannot replicate*.
